# On the Squalene Content of CV Chondrolia Chalkidikis and Chalkidiki (Greece) Virgin Olive Oil

**DOI:** 10.3390/molecules26196007

**Published:** 2021-10-03

**Authors:** Aspasia Mastralexi, Maria Z. Tsimidou

**Affiliations:** 1 Laboratory of Food Chemistry and Technology, School of Chemistry, Aristotle University of Thessaloniki (AUTH), 54124 Thessaloniki, Greece; amastral@chem.auth.gr; 2 Natural Products Research Center of Excellence (NatPro-AUTH), Center for Interdisciplinary Research and Innovation (CIRI-AUTH), 57001 Thessaloniki, Greece

**Keywords:** virgin olive oil, Chondrolia Chalkidikis cultivar, Chalkidiki cultivar, squalene, maturity index, stability study, HPLC-UV, GC-FID

## Abstract

This work is a continuation of efforts to establish the nutritional profile of virgin olive oil (VOO) from cv. Chondrolia Chalkidikis and Chalkidiki and to strengthen its positioning in the global VOO landscape. VOOs produced at an industrial scale in different olive mills of the Chalkidiki (Greece) regional unit as well as VOOs obtained at the laboratory scale from drupes of different maturity stages for four consecutive harvesting years were examined for their squalene (SQ) content using both HPLC and GC procedures. The mean values of SQ were found to be 4228 (HPLC) and 4865 (GC) mg/kg oil (*n* = 15) and were of the same magnitude as that in VOOs from cv Koroneiki (4134 mg/kg, *n* = 23). Storage of VOOs in the dark at room temperature for 18 months indicated an insignificant mean SQ content loss (~2%) in comparison to a mean loss of 26% for alpha-tocopherol content. This finding strengthens our view that SQ does not act as a radical scavenger that donates hydrogen atoms to the latter. The four consecutive harvest years studied indicated a clear declining trend in VOO SQ concentration upon olive ripening. To our knowledge, this is the first systematic work concerning the SQ content of Chondrolia Chalkidikis and Chalkidiki VOOs.

## 1. Introduction

Human health is strongly associated with daily dietary patterns and overall lifestyle. The Mediterranean Diet, which is recognized by UNESCO as an intangible heritage of humanity, is one of the most attractive dietary patterns regarding the regular consumption of an array of functional, tasty foods among which is virgin olive oil (VOO). It is thus considered a key component against various chronic degenerative diseases [1]. Its nutritional and health value is attributed, except for the high concentration of monounsaturated fatty acids (MUFA), to the ideal ratio of alpha-tocopherol (α-T)/polyunsaturated fatty acids (PUFA) content, the presence of minor compounds such as the polar phenol fraction rich in rare secoiridoids (hydroxytyrosol (Htyr), tyrosol (Tyr) and respective bound forms) [2], and last but not least, the presence of squalene (SQ) and related compounds [3].

**Figure 1:**
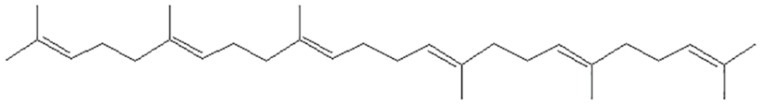
Chemical structure of squalene (SQ, C_30_H_50_, 2,6,10,15,19,23-hexamethyltetracosa-2,6,10,14,18,22-hexaene).

Squalene (Figure 1), the major constituent of the VOO minor compound fraction, is a known biological responses modifier regarding oxygen, the immune system, and steroid synthesis that confers antiaging, anti-inflammatory, and hypocholesterolemic activities [4]. Its detoxifying capability against xenobiotic products concomitantly with ex vivo results associated with cardiovascular diseases supports a possible protective role of SQ against oxidative stress and free radicals and shows the high potential of this molecule for use in nutrition, pharmaceuticals, cosmetics, and medicine [5].

The main source of SQ used to be the liver oil of certain deep-sea sharks in which its content may reach 80% [6]. Nevertheless, due to ecological reasons, more sustainable sources are mandatory. To this view, VOO prevails in comparison to other plant oils, having a mean concentration of ~5000 mg/kg [6] that seem to be cultivar and origin dependent [7]. The maturity stage of olive drupes is also an influential factor but, so far, the results [8,9,10,11,12,13,14] are controversial in certain cases [15]. It should be mentioned that the concomitant decrease in SQ and the increase in the sterol and total triterpenoid content reported for Meski (Tunisian) olives [16] was interrogated for its validity some years later [11]. SQ intake may range from 200 to 400 mg/day in Mediterranean countries like Greece where the consumption of VOO exceeds 12 kg per capita annually. Considering that its absorption is ~60–80% [15], this high intake is a very interesting fact compared with the respective intake in northern European countries or the United States, which is ~10 fold lower [15,17]. Therefore, SQ can be postulated as one of the most promising functional ingredients of the Mediterranean Diet.

In a previous study [18], we documented Greek VOO cv Chondrolia Chalkidikis and Chalkidiki as a product that can be marketed using three health claims authorized by the EU legislation for this precious natural juice (i.e., for ‘oleic acid’ (C18:1), ‘vitamin E’, and ‘polyphenols’). Health claims are a legal tool (Regulation (EC) No 1924/2006) [19] that help the promotion of high-quality VOOs among informed consumers. To date, no health claim has been released for SQ by the EU authorities. One rejected application ‘due to insufficient scientific documentation was found in the EU Register on Nutrition and Health claims for ‘squalene idrocarburo’ that ‘acts as an antioxidant and protects the skin from damages produced by UV rays [20]. Among the pieces of information, an application should contain solid information on the characteristics of the food/constituent for which the claim is made. Evidence of aspects that may influence the claimed effect (composition, physical and chemical characteristics, manufacturing process, stability) and the performance of a measurement method is also required [21].

Data for SQ content in VOOs from Greek cultivars are limited and not systematic. Kalogeropoulos and Tsimidou [15] reviewed relevant literature up to 2014 and reported a wide range of content (2000–5858 mg/kg) for commercial oils and oils from Mavrolia and Koroneiki cultivars from Peloponnese. Recently, Martakos et al. [22] reported that VOOs from five islands of the Northeastern Aegean Region in the 2017/18 harvesting year presented SQ content within the range 635–6344 (mean value 2195 ± 831 mg/kg, *n* = 452). Their results indicate certain trends but mainly point out the need for a systematic study of Greek olive cultivars regarding SQ evolution and dependence on cultivar and maturity stage. Mikrou et al. [23] analyzed 68 samples from two cultivars and three different regions during the harvesting year 2018/19 and found that cv. Koroneiki VOOs presented a higher content (8576 ± 1546 mg/kg, *n* = 59) than those of the cv. Kolovi (5440 ± 822 mg/kg, *n* = 9). Differences in the SQ content in VOOs of the cv Koroneiki were observed among the four regional units of Crete. The highest values ever reported for Greek VOOs were found for samples originating from the Rethimnon regional unit. Yet, differences in VOO SQ content due to the cultivar are not easy to substantiate because of the limited data available. Moreover, the fact that different analytical protocols are used to produce compositional data indicates the lack of a validated protocol to support commercial needs and official control. It is worthy to mention that various chromatographic and spectroscopic protocols, involving sample pretreatment or not, can be found in the literature [6,24,25,26,27,28].

The present work is a continuation of our efforts to establish the nutritional profile of the VOO from cv. Chondrolia Chalkidikis and Chalkidiki and to strengthen its positioning in the global VOO landscape. VOOs produced at an industrial scale in different olive mills of the Chalkidiki (Greece) regional unit were examined for their SQ content just after sampling and during storage to examine the stability of this constituent. Analysis was performed using both HPLC and Gas Chromatography. Moreover, VOOs obtained at the laboratory scale from drupes of these cultivars at different maturity stages for four consecutive harvesting years were also examined for their SQ content to establish natural variability. Results were also discussed with regard to those for VOOs from the major Greek olive cultivar, Koroneiki. To our knowledge, this is the first systematic collection of data to be published for the squalene content of Chondrolia Chalkidikis and Chalkidiki VOO.

## 2. Materials and Methods

### 2.1. Chemicals and Other Materials

Squalene (SQ) (>98%) was sourced from Sigma–Aldrich (Steinheim, Germany) and pyrogallol (>98%) was from Fluka Chemie GmbH (Buchs, Switzerland). Sodium chloride (99.8%) and potassium hydroxide were products of Panreac Quimica (Barcelona, Spain). For SQ-HPLC analysis, acetonitrile (HPLC, 99.9%), methyl acetate (HPLC, 99.8%), and acetone (HPLC, 99.8%) were obtained from ChemLab (Zeldegem, Belgium); *n*-hexane (proanalysis) was from Merck (Darmstadt, Germany) and absolute ethanol was from Riedel de Haën (Seelze, Germany). Other standards, solvents, reagents, and chromatographic materials were of the appropriate grade from various suppliers and have been described in detail in our recent article [18]. Polyvinylidene fluoride (PVDF) membrane filters (0.22 µm) and cellulose acetate membrane filters (0.45 µm) were from Schleicher & Schuell, (Dassel, Germany).

### 2.2. Olive Oil Samples

Representative VOOs from fifteen (15) olive mills of the regional unit of Chalkidiki were obtained to cover the majority of the cv. Chondrolia Chalkidikis and Chalkidiki production in the harvest year 2016/17 (November–December 2016). The sampling zone is shown in Supplementary Materials Map S1. Table S1 shows relevant metadata for the 15 olive mills and Table S2 shows the quality and compositional data reported earlier in [18] for the same samples. In the harvesting years 2017/18, 2018/19, 2019/20, and 2020/21, olives (cv. Chondrolia Chalkidikis and Chalkidiki) were sampled at different maturity stages from 3 different olive groves (OG) in the area of N. Triglia (Chalkidiki, Greece) indicated by a cycle in Map S1. VOOs were extracted under cold conditions using an Abencor laboratory olive mill (MC2, Ingenierías y Sistemas, Seville, Spain). Exact details for the sampling design at different maturity stages can be found in [18] together with data for oil yield and quality characteristics of the oils obtained. Briefly, 10 olive trees in the center of each OG were selected in terms of their similar fruit load and fruit maturity index (MI). Sampling started in mid-September (15/9) and was then repeated approximately every 1–2 weeks until 14/11. For each sampling, 1.5–2 kg fruits were hand-harvested from 3 levels around each tree canopy between 09.00 and 11.00 am. In each harvesting year, the producer harvested the olives on the date he considered technologically optimum based on his experience (29/10 in 2017; 22/10 in 2018; 16/11 in 2019; and 29/10 in 2020). The olives were transferred to a two-phase olive mill. The produced VOOs were coded Mill17_29/10, Mill18_22/10, Mill19_16/11, and Mill20_29/10, respectively. A portion of the same batch of olives was used for the laboratory-scale extraction of VOO the same date.

VOO samples (cv Koroneiki) were acquired directly from mills and producers, who guaranteed their authenticity. The VOOs were from different locations of Greece, mainly Peloponnese and Crete (*n* = 23, the harvesting year 2016/17).

All samples were stored at −22 °C until analysis.

### 2.3. HPLC-UV Determination of SQ

SQ content was determined after alkaline saponification of the oil by RP-HPLC at 208 nm as described by Grigoriadou et al. [24]. Briefly, 0.1 g of oil was added in a 25 mL glass stopped tube followed by the addition of 3 mL KOH (600 g/L). Then 2 mL ethanol and 5 mL of an ethanolic pyrogallol solution were added (60 g/L). After alkaline saponification at 75 °C for 30 min, 15 mL of the NaCl solution (10 g/L) was added and the mixture was extracted twice with 15 mL of *n*-hexane/ethyl acetate (9:1, *v/v*). After evaporation of the organic phase, the dry matter was diluted in acetone.

Separation was achieved on a C18 column (2504 × 4.6 mm i.d.; 5 mm) (Macherey-Nägel, Düren Germany) maintained at 26 °C on an LC 20AD liquid chromatography (Shimadzu Corporation, Kyoto, Japan) equipped with an SPD-10AV UVVIS detector (Shimadzu Corporation, Kyoto, Japan) using acetonitrile (1.2 mL/min). The injection volume was 10 mL. SQ external calibration curves (10–250 mg/L) were used for quantification. Samples were analyzed in duplicates (CV% = 2.0, *n* = 5).

### 2.4. GC-FID Determination of SQ

SQ was co-determined with FAMEs by GC-FID under the conditions proposed in Regulation (EEC) No. 2568/91 [29]. Transesterification took place using a methanolic solution of potassium hydroxide at room temperature. A capillary, TR-FAME column (60 m × 250 μm i.d., 0.25 μm) (ThermoScientific, Bellefonte, PA, USA) was used. The separation conditions were as follows: Carrier gas: Helium (1.1 mL/min), the injector and detector temperature were set at 240 °C, and the injection volume was 2 μL (split ratio 50:1). The temperature was programmed at 100 °C for 5 min, raised from 100 to 240 °C within 15 min, and held constant at 240 °C for 40 min. The identification of SQ was based on the retention time recorded for the SQ standard. Quantification was carried out using appropriate external calibration curves. Samples were analyzed in duplicate (CV% = 5.4, *n* = 5).

### 2.5. Other Analyses

The determination of official quality parameters was accomplished according to Regulation (EEC) No. 2568/91 and its amendments [29]. Moreover, alpha-tocopherol (α-T) content, total polar phenol (TPP) content, and total Htyr and Tyr content were determined using the materials and methods described in [18]. Briefly, the normal-phase high-performance liquid chromatography method was used for the determination of α-T after sample dilution in *n*-hexane/2-propanol (99:1 *v/v*). TPP content was determined colorimetrically using the Folin–Ciocalteu reagent and total Htyr and Tyr content after acidic hydrolysis of the polar fraction (1 M H_2_SO_4_; incubation at 80 °C) followed by UHPLC chromatographic analysis.

### 2.6. Storage Experiment

Different series of aliquots of the 15 VOOs from Chondrolia Chalkidikis and Chalkidiki were stored in brown bottles without headspace in the dark at room temperature (average temperatures of 17 and 26 °C in September–February and March–August, respectively) and were examined for different parameters just after delivery of the samples (time zero) and after 6, 12, and 18 months of storage.

### 2.7. Statistical Analysis

The SQ content values at different time intervals obtained for VOOs (*n* = 15) were subjected to analysis of variance (ANOVA) using R version 4.0.2 (R Core Team, Vienna, Austria). The effect of storage time (months) was modeled separately for SQ values obtained by HPLC-UV and GC-FID, with a significance threshold at *p* < 0.05. VOO quality indices values obtained for cv. Chondrolia reported in our previous publication [18] (Table S2) and those for cv. Koroneiki presented in Table S3 were subjected to principal component analysis using R.

## 3. Results and Discussion

### 3.1. VOO cv. Chondrolia Chalkidikis/Chalkidiki Produced at Industrial Scale

Table 1 summarizes the data for the SQ content of the representative samples collected from the 15 mills shown in Map S1 using a dedicated RP-HPLC in-house-validated method [24] and by the official gas chromatographic protocol for the % FAMEs composition of olive oil adjusted for the concomitant determination of SQ. Unluckily, until now, the EU’s relevant bodies have not validated the GC-FAME protocol for SQ determination using an external calibration curve. As it can be seen in Table 1, SQ content, using both methods, was found to be of the same magnitude at all storage intervals. ANOVA results indicated that the GC protocol is reliable and could be adopted by the EU authorities as an official procedure, taking into account that the step of triacylglycerol transesterification is already validated. SQ is a rather stable molecule under ambient temperatures in the dark as it was also found in our previous studies for both VOO and model systems (stripped olive oil containing squalene and alpha-tocopherol) [30]. SQ stability was remarkable in all 15 samples irrespective of their commercial category. The data for SQ stability presented here for the 11 extra VOOs strengthen our assumption that this compound does not protect α-T from oxidation [30] as erroneously has been hypothesized in other studies [31]. Indeed, the mean loss for SQ was ~2% (18-month storage) whereas the mean loss for α-T (Table S4) reported earlier by Mastralexi and Tsimidou [18] was ~26% for the same 11 extra VOOs.

**Figure 2:**
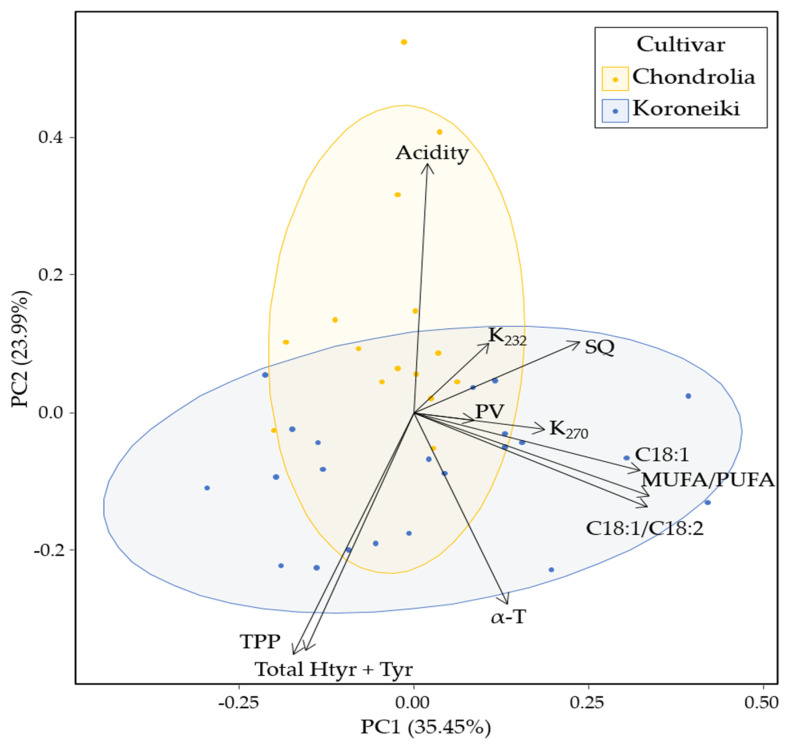
Principal component analysis (PCA) biplot of VOOs from cv. Chondrolia (*n = 15*) and cv. Koroneiki (*n = 23*). The scores were plotted for PC1 and PC2. The proportion of variance explained by each PC is shown in parentheses. The variables used were acidity (% oleic acid), PV (meq O_2_/kg), K232, K270, total polar phenol (TPP), total hydroxytyrosol and tyrosol (Total HTyr + Tyr), α-Tocopherol (α-T), squalene (SQ), oleic acid (C18:1) content and C18:1/C18:2 (linoleic acid) ratio, and MUFA/PUFA ratio. MUFA: Monounsaturated fatty acids; PUFA: Polyunsaturated fatty acids.

The quality indices of VOOs from cv. Chondrolia were compared with those from cv. Koroneiki, the major Greek olive-oil-producing cultivar (Table S3). In total, 23 VOOs obtained from producers and olive mills that guaranteed their botanical origin to be cv Koroneiki were also analyzed for their SQ content and included in the same table. These oils were of the same harvesting period as the oils shown in Table 1 of the present study. All of the samples that were of the commercial category ‘extra’, based on the values of free acidity and K_232_ and K_270_ values, had a high level of antioxidants, and all but one (Koroneiki 14) fulfilled the prerequisites for bearing the authorized health claim for ‘olive polyphenols’ as discussed in [18]. Principal component analysis (PCA) was performed to compare the quality profiles of VOOs from both cultivars (Figure 2).

The first principal component (PC1) explained 35.45% and the second PC explained 23.99% of the total variation. Observations belonging to both cultivars appeared to be clustered together and were not separated on PC1. PC2 appeared to partly separate the two cultivars, probably due to the high acidity values of certain cv Chondrolia samples. VOOs from cv. Koroneiki presented higher loadings for TPP, Total HTyr + Tyr, α-T, C18:1, C18:1/C18:2, and MUFA/PUFA. Indeed, fatty acid compositional data indicated a wider variability of the ratios C18:1/C18:2 (mean value 10.7) and MUFA/PUFA (mean value 9.8) than the respective ones observed for cv Chondrolia Chalkidikis and Chalkidiki VOOs (C18:1/C18:2 and MUFA/PUFA (mean values of 9.9 and 9.2, respectively) [18]. Both cultivars presented similarities regarding PV, K_232_, K_270_, and SQ content. In particular, SQ content was mainly correlated with PC1 and did not appear to drive any potential differences regarding cultivar. According to the data shown in Table S4, the mean value of SQ content for cv Koroneiki VOOs (mean value 4134 mg/kg), as determined by GC-FID, was of the same order as the one found for the cultivars under study using the same analytical procedure. This mean content was ~1/2 of that (8576 ± 1546 mg/kg, the harvesting year 2018/19) reported by Mikrou et al. [23] for VOOs from cv. Koroneiki using a similar GC protocol. Nevertheless, SQ content was almost two-fold higher than the one reported by Martakos et al. [22] for cv Koroneiki VOOs (the harvesting year 2017/18) from olive groves grown in the Northern Aegean Island of Lesvos and of a similar magnitude to that reported by [10] for the harvesting years 2000 and 2004 (Messinia, Peloponnese). Much wider were the mean concentrations of SQ observed for VOOs obtained from different Tunisian cultivars—young or old trees, irrigated or rain-fed [8,9,12,13,14,16]. Analytical procedures vary among publications, so it seems important that the International Olive Council recommend a dedicated analytical procedure for this precious bioactive compound that can add value to the consumption of VOO. The removal of the analytical factor effect would facilitate comparisons of published data and the understanding of natural variability.

### 3.2. VOO cv. Chondrolia Chalkidikis/Chalkidiki Produced at Laboratory Scale

This is the first study on the evolution of SQ in VOO from drupes with an increasing maturity index for so many consecutive years, i.e., 4 years, thus incorporating any climatic differences and biennial bearing effects into natural variability. Available literature data are mostly obtained for one harvesting period. Agronomic practices were the same throughout the 4-year study. The olives were selected using the same calendar every harvesting period, the maturity index (MI) was assessed, and, then the olives were extracted within 2 days max after harvesting. The date on which the producer harvested olives for oil production at an industrial scale was based on his experience of the ‘technological optimum’ stage, and oil was also extracted from the same batch of olives at laboratory scale for comparison. Table 2 summarizes the data for the harvest date, the MI values, and the results for squalene content obtained by two different analytical protocols. The maturity stage negatively affected SQ content as it was observed for all harvesting periods, but the fluctuation within each harvesting year presented a different trend. Results using the two analytical procedures differed within the range of 1–2 standard deviations of the two procedures, indicating that there was no dramatic influence between the two sets of data as also found for the results in Table 1.

## 4. Conclusions

The examination of VOOs from cv Chondrolia Chalkidikis and Chalkidiki for SQ content indicated similar mean values to those observed for VOOs from the cv. Koroneiki, the major Greek oil-producing cultivar. PCA verified this observation. Storage of VOO under appropriate conditions (dark, room temperature, 18 months) did not affect the content of this compound negatively. Considering that, recently, we reported that the best results in terms of the content of oleic acid, α-T, and polar phenolic compounds content in VOO from cv Chondrolia Chalkidikis and Chalkidiki were attained for olives at a maturity index of ~2 [18], it was interesting to find that the same applied for SQ content accumulation. Results from these four consecutive years studied can prove useful to producers and the olive industry in the Chalkidiki regional unit to organize the sector on a scientific basis. These data can be used in the near future regarding a health claim authorization for SQ.

## Figures and Tables

**Table 1:** Squalene (SQ) content of VOOs (mg/kg oil) during the 18-month storage in the dark at room temperature, determined by HPLC-UV and GC-FID. VOOs cv. Chondrolia Chalkidikis and Chalkidiki (the harvesting year 2016/17) were collected from the main olive mills of the regional unit of Chalkidiki.

SQ mg/kg Oil HPLC-UV					SQ mg/kg Oil GC-FID				
Storage Time (Months)									
Sample	0	6	12	18	Sample	0	6	12	18
Extra VOOs									
1	3538	3877	3661	3718	1	4134	4250	3831	3832
2	4525	4510	4569	4471	2	5026	5298	5013	4723
3	4525	4752	4828	4912	3	5284	5538	5410	5339
4	4684	4838	4990	4962	4	5242	5196	5567	5102
5	2762	2844	2703	2715	5	4092	3256	3226	3198
6	3835	3472	3677	4177	6	4488	4356	3247	3647
7	4625	4933	4826	4525	7	6367	5495	5480	4632
8	4744	4708	4764	4907	8	5799	4773	4421	4980
9	3442	3520	3219	3444	9	4725	4773	4086	3347
10	2970	2921	2992	2916	10	3157	3734	3229	2286
11	4726	4668	4671	4737	11	5219	3122	5573	4952
VOOs									
12	4219	4145	4157	4190	12	3789	5528	4719	4777
13	5967	5101	5068	5197	13	6060	5757	5521	5263
14	4119	3835	3720	3616	14	4718	4257	4272	4457
15	4742	4075	3600	3869	15	4875	4512	4205	4433
Mean Value (*n* = 15)	4228 ± 817	4147 ± 723	4096 + 779	4157 ± 762	Mean Value (*n* = 15)	4865 ± 863	4656 ± 836	4520 ± 886	4331 ± 883
SEM ^1^	199					224			
*p-*Value ^2^	0.973					0.395			

All measurements are mean values of duplicate injections. ^1^ Standard error of the mean. ^2^ Estimated by ANOVA with storage time as a factor.

**Table 2:** Variability in the squalene (SQ) content (mg/kg oil) of VOOs extracted from olives cv. Chondrolia Chalkidikis and Chalkidiki at different stages of maturity for four consecutive harvesting years.

	2017/18			2018/19			2019/20			2020/21		
		SQ mg/kg			SQ mg/kg			SQ mg/kg			SQ mg/kg	
Harvest Date	MI**	LC-UV*	GC-FID*	MI**	LC-UV*	GC-FID*	MI**	LC-UV*	GC-FID*	MI**	LC-UV*	GC-FID*
**VOOs Produced at Laboratory Scale**												
15/9	0.9 ± 0.0	4640	3618	0.9 ± 0.1	4327	3757	0.8 ± 0.0	4064	3116	1.0 ± 0.0	3028	2753
3/10	-	-	-	1.3 ± 0.2	4019	4196	1.4 ± 0.1	2903	2388	2.2 ± 0.5	2836	2244
8/10	2.0 ± 0.4	3466	3887	1.7 ± 0.6	3885	3930	2.1 ± 0.1	2913	2468	-	-	-
15/10	2.7 ± 0.5	3503	5431	2.3 ± 0.7	3378	3145	3.1 ± 0.3	2643	2003	-	-	-
22/10	3.2 ± 0.4	3246	3807	2.4 ± 0.5	2897	2946	3.6 ± 0.2	2704	2307	3.3 ± 0.4	4265	2626
29/10	3.3 ± 0.5	3101	3003	-	-	-	3.8 ± 03	2898	1868	3.8 ± 0.3	2104	1604
16/11	3.7 ± 0.4	3144	3124	-	-	-	4.0 ± 0.2	3559	2074	-	-	-
**VOOs Produced at Industrial Scale**												
Mill18_22/10				2.4 ± 0.5	3155	3762						
Mill17_29/10 Mill 20_29/10	3.3 ± 0.5	3621	5731							3.8 ± 0.3	2347	2028
Mill19_16/11							4.0 ± 0.2	3889	3267			

* Mean values (*n* = 2); ** Mean value ± standard deviation (*n* = 3). Mill codes as in [18].

## Data Availability

The data presented in this study are available on request from the corresponding author.

## Supplementary Materials

The following are available online, Map S1: Olive mills in Chalkidiki regional unit involved in the sampling design, Table S1: Loca-tion, extraction system, production capacity, and malaxation temperature of the olive mills in-volved in the sampling design, Table S2: Quality indices, total polar phenol (TPP), total hydroxy-tyrosol (Htyr) and tyrosol (Tyr), α-Tocopherol (α-T), squalene (SQ), oleic acid (C18:1) content and C18:1/C18:2, MUFA/PUFA ratio in VOOs cv. Chodrolia Chalkidikis and Chalkidiki obtained during the harvesting year 2016/17, Table S3: Quality indices, total polar phenol (TPP), total hy-droxytyrosol (Htyr) and tyrosol (Tyr), α-Tocopherol (α-T), squalene (SQ), oleic acid (C18:1) con-tent and C18:1/C18:2, MUFA/PUFA ratio in VOOs cv. Koroneiki obtained during the harvesting year 2016/17, Table S4: α-Tocopherol (α-T) content in Extra VOOs cv. Chondrlia Chalkidikis and Chalkidiki obtained during the harvesting year 2016/17 and % loss of content during the 18-month storage in the dark at room temperature reported in [18].

## Sample Availability

Not available.
