# Supplementary material for: On the Squalene Content of CV Chondrolia Chalkidikis and Chalkidiki (Greece) Virgin Olive Oil
Source: Molecules. 2021 Oct 3;26(19):6007. doi: 10.3390/molecules26196007 (PMC8512499; doi:10.3390/molecules26196007)
Supplement: Supplementary file 1 [file molecules-26-06007-s001.zip › molecules-1403375-supplementary.pdf]

molecules

MDPI

Supplementary Material

On the Squalene Content of cv Chondrolia Chalkidikis and Chalkidiki (Greece) Virgin Olive Oil.

Aspasia Mastralexi [1] and Maria Z. Tsimidou [1,2,*]

[1] Laboratory of Food Chemistry and Technology, School of Chemistry, Aristotle University of Thessaloniki (AUTH), 54124 Thessaloniki, Greece; amastral@chem.auth.gr

[2] Natural Products Research Center of Excellence (NatPro-AUTH), Center for Interdisciplinary Research and Innovation (CIRI-AUTH), 57001 Thessaloniki, Greece

* Correspondence: tsimidou@chem.auth.gr; Tel.: +30-2310997796

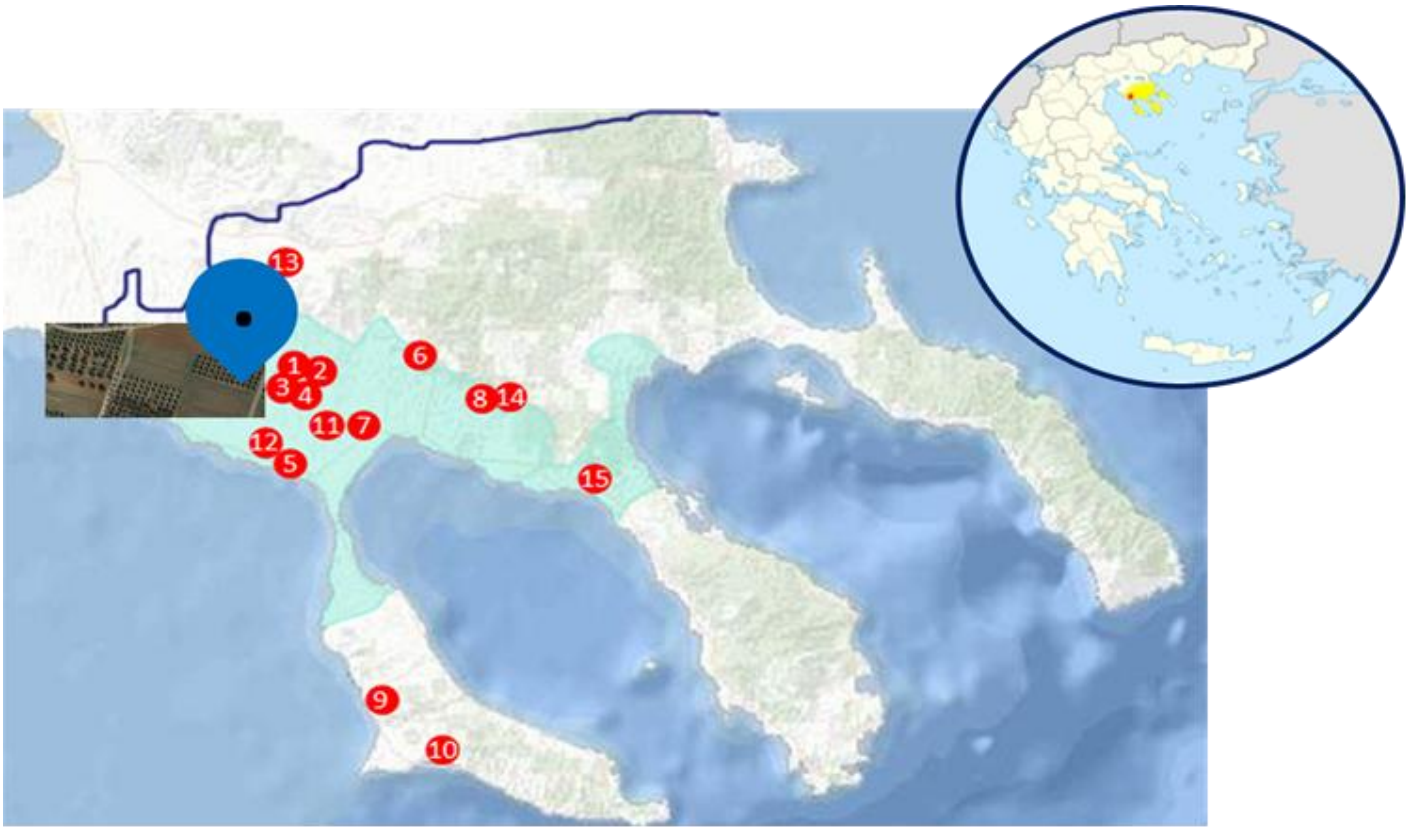

Map S1. Olive mills in Chalkidiki regional unit involved in the sampling design. The shaded light green color area reflects the highest olive growing activity in Chalkidiki. The olive groves (OG) in the area of N. Triglia (Chalkidiki, Greece) indicated by the blue cycle were in the three followed location coordinates: The OG1 (Greek Coordinate System EGSA87/EPSG: 2100): x = 431,298.495, y = 4,461,830.341; OG2: x = 431,360.306, y = 4,461,770.941 and OG3: x = 431,445.678, y = 4,461,792.891.

Table S1. Location, extraction system, production capacity and malaxation temperature of the olive mills involved in the sampling design.

Sample	Olive Mill/ Location	Extraction System	Production Capacity	Malaxation Temperature	Sample	Olive mill/Location	Extraction System	Production Capacity	Malaxation Temperature
1	Olive mill 1/Simandra	three-phase	4 t/h	25–30 °C	9	Olive mill 9/Fourka	three-phase	8 t/h	29–32 °C
2	Olive mill 2/Simandra	two-phase	4 t/h	22–23 °C	10	Olive mill 10/Kalandra	three-phase	2 t/h	-
3	Olive mill 3/Portaria	three-phase	4 t/h	28–33 °C	11	Olive mill 11/Olynthos	three-phase	3 t/h	~27 °C
4	Olive mill 4/Portaria	two-phase	12 t/h	22–23 °C	12	Olive mill 12/Dionisiou	three-phase	2.5t/h	25–28 °C
5	Olive mill 5/N. Moudania	three-phase	2.5 t/h	~38 °C	13	Olive mill 13/Galatista	three-phase	2.5t/h	30–32 °C
6	Olive mill 6/Polygyros	three-phase	2,5 t/h	-*	14	Olive mill 14/Ormylia	three-phase	4 t/h	~38 °C
7	Olive mill 7/Kalyves	three-phase	5 t/h	30 °C	15	Olive mill 15/Nikiti	three-phase	3 t/h	28–30 °C
8	Olive mill 8/Ormylia	three-phase	2 t/h	-					

*-: data not provided by the mill operator.

Table S2. Quality indices, total polar phenol (TPP), total hydroxytyrosol (Htyr) and tyrosol (Tyr), α-Tocopherol (α-T), squalene (SQ), oleic acid (C18:1) content and C18:1/C18:2, MUFA/PUFA ratio in VOOs cv. Chodrolia Chalkidikis and Chalkidiki obtained during the harvesting year 2016/17 [18].

Samples	Acidity* (% Oleic Acid)	PV* (meqO_2/kg)	K_{232}*	K_{270}*	TPP** mg/kg	Total Htyr + Tyr mg/20g	α-T* mg/kg	C18:1*	C18:1/C18:2*	MUFA/PUFA *
1	0.23	10.5	2.14	0.11	247	5	189	74.8	9.7	9.2
2	0.45	11.7	2.03	0.10	348	8	235	75.0	11.1	10,2
3	0.42	7.8	1.76	0.08	211	3	224	75.1	10.8	10.0
4	0.45	10.0	1.87	0.11	215	4	263	74.2	11.0	10.1
5	0.56	10.1	1.75	0.14	255	8	174	72.3	7.2	6.8
6	0.34	8.2	1.85	0.12	277	7	166	74.9	9.7	9.1
7	0.62	9.4	1.47	0.08	261	7	205	74.5	10.4	9.7
8	0.80	8.4	1.47	0.08	184	4	204	74.4	10.1	9.5
9	0.45	7.6	1.76	0.11	290	6	147	74.9	8.9	8.6
10	0.28	9.1	2.03	0.10	445	8	168	74.1	8.0	7.6
11	0.56	9.2	1.91	0.08	240	9	189	75.1	11.5	10.7
12	0,96	10.6	1.59	0.13	277	4	194	73.4	8.3	7.9
13	2.28	10.1	2.20	0.14	78	2	216	73.6	8.3	7.8
14	2.84	16.1	2.39	0.15	47	2	126	72.7	7.9	7.6
15	1.90	10.5	2,05	0.13	123	4	194	73.7	8.1	8.4

* Mean values (n = 2); The total amount of Htyr and Tyr is calculated as the sum of the mean value of three replicates of total Htyr and mean value of three replicates of total Tyr; the sum is then rounded to the first integer; C18:2: linoleic acid; MUFA: monounsaturated fatty acids; PUFA polyunsaturated fatty acids.

Table S3. Quality indices, total polar phenol (TPP), total hydroxytyrosol (Htyr) and tyrosol (Tyr), α-Tocopherol (α-T), squalene (SQ), oleic acid (C18:1) content and C18:1/C18:2, MUFA/PUFA ratio in VOOs cv. Koroneiki obtained during the harvesting year 2016/17.

Samples	Acidity* (% Oleic Acid)	PV* (meqO_2/kg)	K_{232}*	K_{270}*	TPP** mg/kg	Total Htyr + Tyr mg/20g	α-T* mg/kg	SQ* mg/kg	C18:1*	C18:1/C18:2 *	MUFA/PUFA*
Koroneiki 1	0.39	12.3	1.83	0.13	228 ± 10	7	309	6137	75.0	11.30	10.29
Koroneiki 2	0.62	10.9	1.60	0.12	224 ± 10	7	299	6142	75.2	11.75	10.64
Koroneiki 3	0.34	11.4	1.96	0.15	346 ± 23	11	345	5867	75.3	13.70	12.19
Koroneiki 4	0.39	12.5	1.96	0.15	368 ± 28	8	244	4889	74.3	10.41	9.60
Koroneiki 5	0.45	10.3	1.93	0.12	204 ± 13	5	279	5861	74.6	10.64	9.68
Koroneiki 6	0.39	9.8	1.87	0.11	223 ± 10	5	252	5546	78.5	15.32	13.80
Koroneiki 7	0.23	10.7	1.70	0.12	275 ± 70	7	266	3868	75.9	11.14	10.27
Koroneiki 8	0.28	11.2	1.91	0.12	338 ± 20	9	224	3567	73.4	8.49	7.99
Koroneiki 9	0.39	9.9	1.66	0.12	352 ± 30	8	231	3666	72.7	8.08	7.59
Koroneiki 10	0.28	10.8	2.14	0.12	392 ± 80	10	237	3714	73.8	8.53	7.98
Koroneiki 11	0.56	8.9	1.58	0.08	429 ± 11	10	244	3606	73.3	8.65	8.09
Koroneiki 12	0.34	10.5	1.82	0.10	409 ± 90	13	226	2610	71.8	7.29	6.89
Koroneiki 13	0.34	11.9	0.19	0.12	413 ± 17	13	269	4062	73.1	8.87	8.25
Koroneiki 14	0.45	15.5	1.93	0.13	98 ± 60	3	309	3833	75.8	10.51	9.63
Koroneiki 15	0.51	10.9	2.50	0.16	418 ± 17	13	264	3480	74.7	10.79	9.92
Koroneiki 16	0.51	9.8	1.64	0.12	507 ± 70	12	254	4903	74.9	10.27	9.48
Koroneiki 17	0.51	11.3	2.27	0.15	307 ± 10	9	227	5220	76.6	11.60	10.69
Koroneiki 18 The total amount of Htyr and Tyr is calculated as the sum of the mean value of three replicates of total Htyr and mean value of three replicates of total Tyr; the sum is then rounded to the first integer	0.51	8.6	2.16	0.17	300 ± 17	7	240	4977	76.9	11.94	10.88
Koroneiki 19	0.56	15.1	2.27	0.12	345 ± 11	7	236	3488	70.8	6.89	6.51
Koroneiki 20	0.56	16.9	2.21	0.15	452 ± 26	13	337	3863	72.85	8.49	7.91
Koroneiki 21	0.62	13.8	2.11	0.20	265 ± 8	6	293	5328	78.87	16.16	14.23
Koroneiki 22	0.34	13.0	1.97	0.12	508 ± 5	15	252	3977	73.69	9.24	8.55
Koroneiki 23	0.68	17.7	2.40	0.20	175 ± 7	3	237	4754	78,66	14.74	13.17

* Mean values (n = 2); ** Mean value ± standard deviation (n = 3); The total amount of Htyr and Tyr is calculated as the sum of the mean value of three replicates of total Htyr and mean value of three replicates of total Tyr; the sum is then rounded to the first integer; C18:2: linoleic acid; MUFA: monounsaturated fatty acids; PUFA: polyunsaturated fatty acids.

Table S4. α-Tocopherol (α-T) content in Extra VOOs cv. Chondrlia Chalkidikis and Chalkidiki obtained during the harvesting year 2016/17 and % loss of content during the 18-month storage in the dark at room temperature reported in [18].

Sample	0	18	% loss	Sample	0	18	% loss
1	189	149	21	7	205	140	32
2	235	173	26	8	204	160	22
3	224	164	27	9	147	110	25
4	263	177	33	10	168	114	32
5	174	118	32	11	189	133	30
6	166	139	16				
